# What bereaved parents want health care providers to know when their babies are stillborn: a community-based participatory study

**DOI:** 10.1186/s40359-020-0385-x

**Published:** 2020-02-17

**Authors:** Lynn L. Farrales, Joanne Cacciatore, Christine Jonas-Simpson, Shafik Dharamsi, Jaime Ascher, Michael C. Klein

**Affiliations:** 1 Department of Family Practice, University of British Columbia, Vancouver, Canada; 2 Still Life Canada: Stillbirth and Neonatal Death Education, Research and Support Society, Vancouver, Canada; 3 International Stillbirth Alliance, Bristol, UK; 4 Arizona State University West, 4701 W Thunderbird Road, Glendale, AZ USA; 5 York University School of Nursing, Toronto, Canada; 6 College of Health Sciences, Peter De Wetter Distinguished Professor of Health Sciences, The University of Texas at El Paso, El Paso, USA; 7 Emeritus Professor Family Practice & Pediatrics, Department of Family Practice, University of British Columbia, Vancouver, Canada; 8 Senior Scientist Emeritus, BC Children’s Hospital Research Institute, Vancouver, Canada

**Keywords:** Stillbirth, Perinatal death, Grief, Traumatic grief, Complicated grief, Newborn death

## Abstract

**Background:**

Bereaved parents experience higher rates of depressive and post-traumatic stress symptoms after the stillbirth of a baby than after live-birth. Yet, these effects remain underreported in the literature and, consequently, insufficiently addressed in health provider education and practice. We conducted a participatory based study to explore the experiences of grieving parents during their interaction with health care providers during and after the stillbirth of a baby.

**Methods:**

This community-based participatory study utilized four focus groups comprised of twenty-seven bereaved parents (44% fathers). Bereaved parents conceptualized the study, participating at all stages of research, analyses, and drafting. Data were reduced into a main theme and subthemes, then broad-based member checked to ensure fidelity and nuances within themes.

**Results:**

The major theme that emerged centered on provider acknowledgement of the baby as an irreplaceable individual. Subthemes reflected 1) acknowledgement of parenthood and grief, 2) recognition of the traumatic nature of stillbirth, and 3) acknowledgement of enduring grief coupled with access to support. It was important that providers realized how grief was experienced within health care and social support systems, concretized by their desire for long-term, specialized support.

**Conclusions:**

Both mothers and fathers feel that acknowledgement of their baby as an individual, their parenthood, and their enduring traumatic grief by healthcare providers are key elements required in the process of initiating immediate and ongoing care after the stillbirth of a baby.

## Background

There exists much stigma, social shame and marginalization, associated with parental grief after the stillbirth of a baby that often arises from with interactions with medical providers, family and friends, colleagues, and even the general public [1, 2]. Despite that 2.6 million babies are stillborn worldwide each year, and about the same number of neonates die annually [2–4], much more remains to be learned about the effects of a baby’s death. There has been minimal attention on parental grief after stillbirth in health care curricula leading to inadequate support for bereaved families both during the acute crisis of a baby’s death and also in long-term care [5–8].

Unique from the grief following the death of an older child, the grief after a baby is stillborn is remarkably under estimated, disenfranchised, and misunderstood [1, 5, 6]. Stillbirth is associated with poor psychological outcomes, and grieving parents experience higher rates of emotional distress and post-traumatic symptoms than non-bereaved parents [2, 9]. The role of healthcare providers (HCPs) during the delicate period leading up to and following the stillbirth of a baby is critical. However, little is known about the experiences and needs of bereaved parents after stillbirth from their perspective, especially fathers who are often underrepresented [10].

Prior research on stillbirth and perinatal death has largely examined the impact of HCPs [5], adverse psychosocial outcomes [11], epidemiological factors [3], and prevention [12] in studies designed solely by researchers. This participatory research, a qualitative research design [10, 11] that enabled bereaved parents and their family members to play a key role in every aspect of the research [12], explored the experiences of bereaved parents. Bereaved fathers were remarkably well represented in this study, consisting nearly half the sample, unlike in prior research. This exploration was to gain a deeper understanding of their interactions with HCPs in the period prior to and after stillbirth. This is of great import to the body of literature around the death of a baby because of the uniquely high representation of fathers in the sample and because prior studies have not utilized participatory research with this vulnerable population.

## Methods

Because of the vulnerability of this group, we employed a qualitative participatory research design [13–16] that is respectful of participants’ wisdom and expertise, empowers their autonomy and agency, and gives voice to their experiences. This type of approach emphasizes depth in research and is considered more ethical, culturally appropriate, and community-based. In any type of disenfranchised, or socially marginalized, grief experience, oft marked with stigma and isolation, participatory research can provide an important insight into how to best realize the agency of parents [16]. Consistent with the tenants of participatory research, bereaved parents and their family members conceptualized the study, set the research question and design, conducted focus group discussions, assisted in analyzing data, and participated in the writing and editing of this paper [13–16].

Participants were recruited from a cohort of bereaved parents who participated in a two-day workshop on the topic of grief after stillbirth. Successful recruitment of fathers may have been due to the involvement of a local organization’s board that was well-represented by fathers. Inclusion criteria included bereaved parents, 19 years of age or older, who experienced the stillbirth of a baby. The study was approved by the principal investigator’s Institutional Review Board at the University of British Columbia. Written informed consent was obtained from all research participants prior to participating in the study.

### Focus groups and participants

Four, 90 min long focus groups were conducted simultaneously by facilitators trained in sensitive qualitative research methods who were bereaved parents or their bereaved family members. A trained research assistant (non-bereaved) was asked to attend the groups to take notes. Twenty-seven parents participated, comprising 12 fathers and 15 mothers, with a mean age of 39 (Table 1). The time since the death of the baby ranged from < 2 months to 20 years, however, 63 % reported a loss in the past four years. Two of the focus groups consisted of only mothers, with six participants in each group; a third group consisted of a mix of mothers and fathers (n = 6), and the fourth focus group had only fathers (n = 9) (Table 2). Twenty-five of the 27 parents reported their babies were stillborn (See Table 1) (two participants reported having multiple losses, both stillbirth and neonatal death). Couples had a choice to be in a focus group with their partners or to be in different groups; to maintain anonymity, cross-referencing of partners across focus groups was not done.

**Table 1:** Participant Demographic and Background Information

Demographic Item	
Age	**Mean Age 39 (27–58)**
Male	12 (44%)
Female	15 (56%)
Married	23 (85%)
College educated	23 (85%)
Household income >/=$75,000	17 (63%)
Organized religion	10 (37%)
Spiritual	16 (60%; 15% NR)
Living children	9 (33%; 7% NR)
Formal grief support received (therapist/support group)	24 (89%)
**Time since stillbirth of baby**	**Number of Participants**
≤ 2 months	5 (19%)
≤ 1 year	2 (7%)
< 2 years	7 (26%)
2–4 years	6 (22%)
18–20 years	3 22% (NR)

**Table 2:** Themes and Subthemes

Theme	
*Acknowledgement of baby as an irreplaceable individual*	
Subthemes	
*1. Acknowledgement of parenthood and parental grief*	
• Finite window to interact with baby’s body	
• Provider compassion toward parents specific to their grief	
• Acts of parenting	
*2. Acknowledgement of trauma*	
• Health care provider and parental knowledge of concept of trauma	
• Provider compassion toward their experience of trauma	
• Environmental factors and systems that mitigate or exacerbate trauma	
*3. Acknowledgement over time and space through access to specialized support*	

### Analyses

The four focus group discussions were audio-recorded and transcribed verbatim. Transcripts were analyzed by first coding each transcript individually and then collectively for distinct concepts and categories of meaning, which were then grouped into themes [13, 14]. To ensure credibility of the analytic process and fidelity to the outcomes of group discussions, the data analysis team kept reflective journals and field notes during the entire course of study. In these entries, team members recorded the experience and process of participation. Reflective notes highlighted personal values and interests that influenced analyses and improved validity procedures [17]. Through member checking, these notes helped each research team member bring to the interpretation of the data a richer understanding of the complex phenomena of bereavement after a baby’s death [17].

Co-investigators shared emergent themes with bereaved parents and HCPs in various community settings. This broad-based member check contributed to the trustworthiness of the findings, while uncovering nuances within key themes, such as the role of HCPs during the finite window of opportunity to interact with a baby after death.

## Results

The research team did not find differences between mothers and fathers in the major themes. That is, both mothers and fathers spoke about their grief and trauma and the need for acknowledgement and support in similar ways. Specifically, the acknowledgement of the baby as an irreplaceable individual was foundational to the themes of acknowledgement of parenthood and parental trauma and grief. In addition, acknowledgement over time and space, that is, the recognition that grief felt infinite and pervasive, present in all aspects of life from home and work to recreation and family events, was also important, buttressed by their desire for long-term, specialized support across systems. Quotes reflected included diverse members of each focus group (FG).

### Acknowledgement of baby as an irreplaceable individual

The major theme that emerged focused on the desire from parents to have their babies acknowledged as irreplaceable individuals.

FG-4: I wish like at the beginning, especially in the hospital environment, they would have identified my daughter as a singular, as my daughter, as my first born as opposed to thinking, “Oh, you guys get healthy and you just go out and do it again and everything will be over, be replaced … ”

Making an effort to interact with the baby who died, just as with any living baby, was a commonly cited example of acknowledgement:

FG-3: And they come up to the baby and ask, ‘Can I see him’ and ‘What’s his name?’ … ‘Oh, he’s so beautiful.’ Love that … You’re a nurse in the maternity ward, you would do that with every live baby …

Participants spoke about terms like “fetus,” “products of conception,” miscarriage,” and even “stillbirth” itself coming across as dehumanizing, distancing and depersonalizing, whereas using the baby’s name was a form of much-needed acknowledgement of the baby and the unique relationship between baby and parent.

FG-3: They asked, ‘Does he have a name?’ and I said his name’s [name], and ever since [indiscernible] they called him by his name. So they gave me that, that personality of a child, like, they acknowledged that I actually had a baby and not just a stillbirth.

Acknowledgement of the baby as physically beautiful by HCPs was also important in assuaging parents’ fears about seeing the baby’s body.

FG-1: I hit him [partner] and said, ‘Look at me,’ ‘cause I didn’t want him to look because, again, I was terrified. And it was … [the midwife] saying, ‘He’s beautiful,’ that I went, ‘Okay, now I want to … ‘ I knew I would hold him, but it was that first look that I was terrified about.

This theme of acknowledging the baby as an irreplaceable individual was a central component to other emergent themes.

### Acknowledgement of parenthood and grief

Participants spoke about how HCPs can facilitate attachment through the recognition of ongoing parental bonds. Acknowledging parenthood was linked to the theme of acknowledging the baby as an irreplaceable individual.

FG-3: I also found when we were there that the nurse came in and they treat you as a patient. And would just come in, take your vital signs and be out. And you’re just there as a number. Whereas other nurses came in and there was a baby. Yes, the baby’s dead. But there is a baby. And I’m still a proud Mom.

FG-4: And then [name] was born and I held her in my arms and here was the greatest challenge … I had this connection to my child. And I don’t know if you guys felt this but, you know, I was really proud. I don’t mean to put a positive spin on the death of my daughter, but I can’t deny … that part was there.

With the acknowledgement of the baby, and of parenthood, came the validation of their profound grief. Participants described other ways in which the depth of their loss was acknowledged. This included experiencing the shared grief and compassion of HCPs.

FG-3: The few nurses … [and] there was one doctor, who were able to cry with us … Those stood out to me. Those are the faces I can still picture … I found that was just so honest and so human that they were able to do that. Just sit there and cry … And that was helpful for me because it was so sad. And it is just a normal reaction*.*

In contrast, another participant described the impact of not being acknowledged by a care provider:

FG-2: … [The doctor was] busy enough that [she] doesn’t need to come and take a look at this baby … That one sticks with me a lot … there was no compassion there whatsoever … from the doctor.

Participants also referred to the finite window of time they had to interact with and ritualize their baby and the important role of HCPs in facilitating this interaction.

FG-4: ‘So how much time do we have?’ And they said, ‘Oh, you can stay as long as you want’ … We actually had [baby] with us in the room like for … two days … We had guidance in terms of … like, ‘Hey, you need to put the baby on ice because … like we need to slow the process down.’ … We had that time and that was really valuable to us.

FG-4: ‘Can I see the baby?’ and she [nurse] just told me, uh, ‘It’s going to look different, it’s going to be hard for you to see [him].’ And, probably had to think about it, yeah, and then just kind of went home … You guys can give me a better understanding … like you had a chance to hold your own babies. But I felt kind of [deprived], like probably this is going to be my whole life.

Participants spoke about the uncertainty of their baby’s whereabouts after birth, during autopsy, and prior to burial or cremation as being a source of distress. One participant spoke about the autopsy as an informed decision a parent would make for a child and described how providers acknowledged their role as a parent by ensuring respect and care for the baby’s body:

FG-4: So, we didn’t want our son to be in some kind of morgue in a different place for two weeks and then the autopsy happens … And they couldn’t answer all those questions. What really helped us agree at some stage was when they said, you know … all these pathologists they are parents themselves and they will honour our child and they will be careful.

One participant described how the provider facilitated acts of parenting for both parents:

FG-4: A few days later we were really worried about how they were going to transport [name] from one hospital to another [for an autopsy] … So … our midwife was really good and she found out like when … our daughter was going to be driven. So, we actually went to the hospital and followed the car from one hospital to the next.

### Acknowledgment of trauma

The words “trauma” and “traumatic” were recurrent in participants’ narratives.

FG-4: The whole experience was chaotic, everything was not as planned. And I didn’t feel that anybody knew what was happening, even the health care professionals … Very traumatic … the image of my life as it was supposed to happen somebody tore it all up into pieces and threw it into the garbage.

Participants also spoke about how some of this trauma can be mitigated merely through its acknowledgment.

FG-2: You know, I didn’t see it as—as a—trauma, um, because nobody ever treated me like it was a trauma … Some education about what fear does to a person would be so helpful. You know, I could have been a lot kinder to myself.

Parents spoke about environmental factors in the hospital that intensified their traumatic experiences, like sounds of babies crying or being born, pictures on walls with images of live babies, visualizing other families with newborn babies and hospital staff interactions that assumed that study participants had a live baby. One participant who spoke about hearing babies while in labor:

FG-2: I was horrified...I said that to the social worker. I said, ‘I can’t believe that you have me here.’ She said, ‘well, there’s been studies … and, you know, it’s good to get mothers integrated with … society right away.’ [Laughter] … [Participant: Oh.] ‘You know, there’s babies in the world and so, you do need to get used to hearing them’ … [Participant: What?] [Participant: Wow.] Like we had this discussion while I’m in labor.

Fathers spoke about how their mobility on the ward and in the hospital placed them in situations that were potentially distressing.

FG-4: Like as a dad, you are the one who walks … you walk along the ward and pass every single door and the door’s open you look inside and see the family and you see the babies and everything you don’t have … I doubt that we’ll ever build a new floor for … bereaved parents.

Participants described the effects of the psychological reminders of loss, underscoring the diversity of participant experiences. For instance, one mother did not wish to be separated from other mothers:

FG-2: So … I don’t know anything about this, just a personal opinion, but I’m thinking if I moved to a different place where the moms of dead babies go to have their dead babies, we’re just getting shoved in a little corner and we’re not integrated with everybody else who—I’m a mother, just like them.

Parents identified the efforts of hospital staff to create a compassionate environment: soundproofing grieving parents’ rooms, respectful signage on doors to indicate a baby has died, removal of posters with newborn photos, closing doors with crying babies, and timing of discharge to avoid seeing other families with their living babies.

FG-3: The hospital I was at also had signs on the door with a teddy bear and a tear. I don’t know if all hospitals have that. But, that helped. The people taking blood and bring [ing] the food, you know, to be sensitive and I think that probably helped prevent a lot of situations. So that helped.

Compassion from HCPs was an essential form of acknowledgement that mitigated the trauma.

FG-3: Just when people are present, you know … .being a healthcare professional, but also to step out of their role and just connect with human to human. [Participant: Yeah. It is the human touch you need.]

### Acknowledgement over time and space through access to specialized support

The presence of specialized support across various systems post-discharge from the hospital represented another form of acknowledgement of the baby, their parenthood, and the breadth and depth their grief. One participant described how phone calls from a provider in the months after her loss were helpful:

FG-2: The community nurse called me, um, every week for the first few months. And then, she called every two weeks … and that just made a huge difference, you know.

Another commented on a support group:

FG –4: I found it really, um, helpful to meet other families, that was the greatest support … .I didn’t have to prove anything.

However, many participants also discussed the difficulty of finding support and resources.

FG-3: … I just felt alone … And I was looking for help. And really, I searched everything … I searched literature and Internet and I wanted to connect to other parents who had lost children … I had that desperate need of hearing about people’s stories and there wasn’t anything … I was thinking ‘Why do I have to look for help?’

Participants spoke of various types of support ranging from follow-up phone calls, medical support, support groups, counseling, and connecting with other bereaved families. While these were important, some participants spoke about the gaps and lack of specialized skills in individual and group settings to meet their needs.

FG-3: You have dead babies and you get ten minutes with a social worker and they give you a package and a call the next day. Yeah. I’m okay.

FG-4: There’s the hospital support and then there’s the long-term support, how to survive in the community … once you leave that hospital you drop off the radar … how do we come up with solutions for these affected bereaved parents?

Several participants also spoke about how postpartum physiology, namely lactation, required acknowledgement from medical providers. Some participants felt supported by their providers, while others were taken by surprise when they began to lactate, and yet others were told that there was nothing that could be done:

FG-3: … it would have been really nice if somebody had told me that my milk was probably going to come in. Two days later that was a complete—shock that I was completely blindsided by.

FG-3: Nope. I was—they didn’t tell me about it. They didn’t give me anything.

## Discussion

Bereaved parents see the acknowledgement of their baby by HCPs as essential to the initiation of immediate and ongoing bereavement care. Their acknowledgement of the baby as an irreplaceable individual emerged as a central theme that underpinned the acknowledgement of parenthood, grief and trauma, and access to acute and long-term specialized support.

Our results are consistent with studies conducted with predominantly bereaved mothers after stillbirth in the United States and Sweden [10, 18, 19]. Grief after the stillbirth of a baby has been described as stigmatized, disenfranchised, and ambiguous [7, 8]. Disenfranchisement is described by Lang [7, 8] as stemming from the contradiction between parental grief after stillbirth and “society’s dismissal” of this grief, while Cacciatore [20] applied Pauline Boss’ theory of ambiguous grief to stillbirth: That is, though the child has died, his or her psychological presence can endure for years. It is the lack of physical evidence verifying the baby’s existence, as well as the marginalization of both the baby and the resultant grief, that incites intense pain and identity distress for parents [20]. Therefore, the central need for acknowledgement of the baby, both in the immediate hospital setting and in the community, may counter the experiences of both societal de-legitimization and role ambiguity.

The core theme of acknowledgement took two forms: individual and systemic. Acknowledgement at the individual level was relational in provider-parent interactions and was found to be important in other studies [21, 22]. Systemic acknowledgement took the form of structural policies and procedures relating to the presence or absence of acute and long-term services, access to specialized counseling and support, and attention to environmental factors within health care settings. Specific policy changes in the care of women and their families should include death education for providers, women-centered education programs focused on relationships, and programs focused on grieving mothers and their families [5].

Trauma, which emerged as a major theme of this study, is supported by prior studies [21, 23]. The role of HCPs in mitigating this trauma through compassionate care was also found to be a recurrent theme. Providers couldn’t lessen the grief around a baby’s death; however, they could provide a sense of care that parents appreciated and that may diminish risk of future adverse psychological outcomes related to traumatic stress.

The literature also suggests that HCPs are greatly impacted when caring for families whose babies are stillborn and require ongoing education and support [1, 5, 24, 25]. The themes that emerged may offer guidance for provider-parent interactions that are more egalitarian, responding more sensitively to the diverse preferences of parents, and this may help providers in knowing they provided more comprehensive and sensitive care. There should be less emphasis on the standardization of care and rigid protocol and checklists; rather the focus should be relationship-based caregiving that underscores the nuances of each family, recognizes their unique culture, and prevents retraumatizing provider reactions [5, 7, 20]. Our findings suggest that acknowledgment of the baby forms the basis of interaction with parents and their future perception of past trauma [22, 25]. Tangible expressions of acknowledgment, like those found in our reported data, also further parental perception of compassionate care and may reduce the likelihood of persistent trauma symptoms.

Finally, prior studies suggest that many bereaved fathers suffer intense psychological and social distress after the stillbirth of their baby, contingent on the degree of attachment. Still, they appear to have limited sources of support, with much of the focus on their partners [26]. This is complicated by societal expectations around emotional expression of men in Western culture [26]. The results of this study demonstrate that some fathers, too, have similar needs as mothers, and HCPs might be more inclusive in their practice and policies.

### Study strengths and limitations

The community-based methodology is novel in this research area. The study team of parents and family members assisted with the development of themes in collaboration with the research team. Another unique strength of this study was that almost one-half of the participants were male. Bereaved fathers are typically in the minority in other studies focusing on parents affected by stillbirth [10, 27]. Discordant grieving styles between fathers and mothers have been associated with relational breakdown and divorce [1]. Our research did not reveal gender discordance with regard to the development of the major themes. It should be noted, however, that although information about self-identified gender and sexuality of the participants was not collected, there remains a predominantly hetero-normative discourse in the narratives. As with the majority of studies in this field, the majority of participants were middle-class and Caucasian. Whether these findings represent the totality of experiences for disadvantaged or minorities families is not known.

## Conclusion

This community based study sought to explore the experiences of parents as they interacted with HCPs after their babies were stillborn. Findings suggest that provider acknowledgement of their baby, their parenthood, and their traumatic grief, both in the immediate aftermath of the death and long-term, is exceedingly important and may affect their lives for years. Consonant with prior research illuminating the importance of staff training and preparedness [28], grieving parents would likely benefit by furthering education on traumatic grief for HCPs, especially models specifically focused on support across various spaces such as hospitals, doctor’s offices, communities, and work places. Because insensitive staff interactions and hospital policies might incite lasting trauma symptoms for parents after the stillbirth of a baby [28] that may endure for years and even decades [5–10, 28], it is imperative that HCPs are well-educated in trauma-informed, compassionate care. This is especially true when considering the finite window of opportunity to interact with the baby’s body and the irreversibility of decisions made in that brief period. In 1977, the British Medical Journal published a scathing harbinger to the medical community about dangerousness of the lack of education and compassion complicated by an abundance of provider hubris and aversion after the stillbirth of a baby: “the danger lies not in the grief and distress; the danger is in bypassing it, thereby promoting a variety of severe psychological complications. The commentary continues to note that “the danger is not merely to the mother but also to her husband, her surviving children, and, worst of all, to the next baby (1157).” [29] Forty-two years later, and some of these very difficult narratives from bereaved parents about their interactions with HCPs persist. Collaboration with parents to identify and implement appropriate services and support in other spaces, during and beyond the acute crisis of a baby’s death, is not only valuable but essential.

Future directions include research in trauma-informed care specific to stillbirth, and research on how to best support HCPs, in light of the emotional and sometimes traumatic nature of their work, advance the provision of relational caregiving. A genuine effort to include populations most affected by stillbirth in bereavement research is warranted. Participatory research methods are recommended to ensure parent and family oriented processes and outcomes in an area of study that remains highly stigmatized.

## Data Availability

Anonymized data may be obtained by contacting the first author: Joanne Cacciatore, PhD, jcaccia@me.com or joanne.cacciatore@asu.edu

## Abbreviation

FG: Focus group
